# Supplementary material for: All Fiber-Optic Immunosensors Based on Elliptical Core Helical Intermediate-Period Fiber Grating with Low-Sensitivity to Environmental Disturbances
Source: Biosensors (Basel). 2022 Feb 6;12(2):99. doi: 10.3390/bios12020099 (PMC8869875; doi:10.3390/bios12020099)
Supplement: Supplementary file 1 [file biosensors-12-00099-s001.zip › biosensors-1574009-supplementary.pdf]

Supplement information

All Fiber-Optic Immunosensors Based on Elliptical Core Helical Intermediate-Period Fiber Grating with Low-Sensitivity to Environmental Disturbances

Junlan Zhong [1,2], Shen Liu [1,2,*], Tao Zou [1,2], Wenqi Yan [1,2], Min Zhou [1,2], Bonan Liu [1,2], Xing Rao [1,2], Ying Wang [1,2], Zhongyuan Sun [1,2] and Yiping Wang [1]

[1] Key Laboratory of Optoelectronic Devices and Systems of Ministry of Education/Guangdong Province, College of Physics and Optoelectronic Engineering, Shenzhen University, Shenzhen 518060, China; zhongjunlan@email.szu.edu.cn (J.Z.); szu_zoutao@163.com (T.Z.); yanwenqi2020@email.szu.edu.cn (W.Y.); minzhou2020@163.com (M.Z.); ssamliu@163.com (B.L.); 2060453012@email.szu.edu.cn (X.R.); yingwang@szu.edu.cn (Y.W.); sunzhongyuan@hotmail.co.uk (Z.S.); ypwang@szu.edu.cn (Y.W.)

[2] Shenzhen Key Laboratory of Photonic Devices and Sensing Systems for Internet of Tings, Guangdong and Hong Kong Joint Research Centre for Optical Fibre Sensors, Shenzhen University, Shenzhen 518060, China

* Correspondence: shenliu@szu.edu.cn

Citation: Zhong, J.; Liu, S.; Zou, T.; Yan, W.; Zhou, M.; Liu, B.; Rao, X.; Wang, Y.; Sun, Z.; Wang, Y. All Fiber-optic Immunosensors Based on Elliptical Core Helical Intermediate-Period Fiber Grating with Low-Sensitivity to Environmental Disturbances. *Biosensors* **2022**, *12*, 99. https://doi.org/10.3390/bios12020099

Received: 12 January 2022
Accepted: 3 February 2022
Published: 6 February 2022

Publisher's Note: MDPI stays neutral with regard to jurisdictional claims in published maps and institutional affiliations.

Copyright: © 2022 by the authors. Licensee MDPI, Basel, Switzerland. This article is an open access article distributed under the terms and conditions of the Creative Commons Attribution (CC BY) license (https://creativecommons.org/licenses/by/4.0/).

1. Fabrication Process of the E-HIPFG

The high-efficiency hydrogen-oxygen flame heating system is consisting of a high-precision rotator, two translation stages, and a hydrogen generator, as schematically represented in Figure S1. In the fabrication process, the elliptical core fiber was melted by hydrogen-oxygen flame heating to write the HIPFG with a rotation rate (Ω) of 2057 rpm. At the same time, the right translational stage V_1 and the left translational stage V_2 were working with velocities of 1.10 mm/s and 1.20 mm/s, respectively.

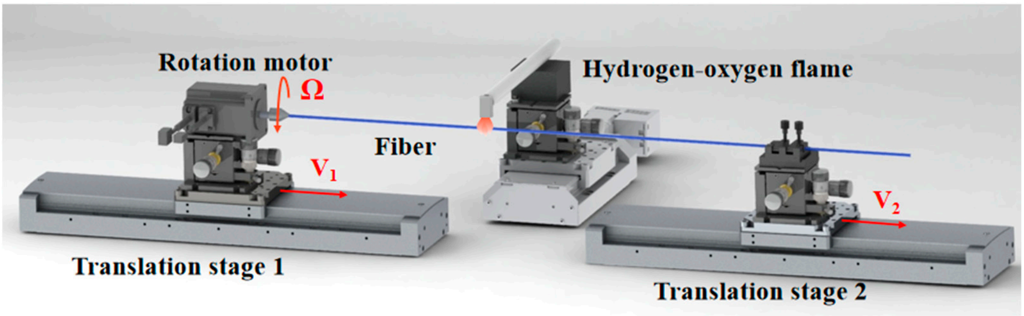

Figure S1. Schematic of hydrogen-oxygen flame heating system.

2. Anti-IgG Immobilization on the E-HIPFG

The transmission spectra of the E-HIPFG at Dip-4 before and after the goat anti-human IgG immobilization are shown in Figure S2. The Dip-4 shows a 0.6 nm of red-shift after the anti-IgG immobilized on the E-HIPFG. The wavelength increase is caused by the rise of surrounding RI and expresses the success of the immobilization [1].

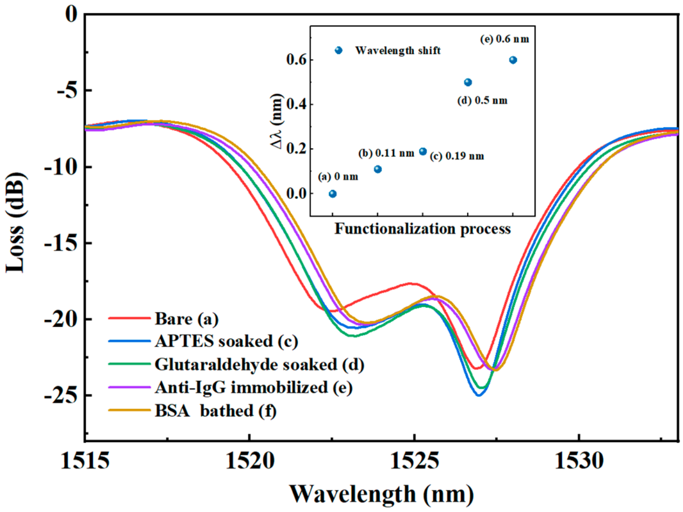

Figure S2. The transmission spectra of the E-HIPFG at Dip-4 in the anti-IgG immobilization process. The inset figure illustrates the wavelength shift of the immobilization process.

Reference

1. Shu, X.; Huang, D. Highly sensitive chemical sensor based on the measurement of the separation of dual resonant peaks in a 100-μm-period fiber grating. *Opt. Commun*. **1999**, *171*, 65–69. https://doi.org/10.1016/S0030-4018(99)00522-2
